# Effectiveness of heat-sensitive moxibustion in the treatment of lumbar disc herniation: study protocol for a randomized controlled trial

**DOI:** 10.1186/1745-6215-12-226

**Published:** 2011-10-13

**Authors:** Mingren Chen, Rixin Chen, Jun Xiong, Fan Yi, Zhenhai Chi, Bo Zhang

**Affiliations:** 1 The Affiliated Hospital of Jiangxi University of TCM, Nanchang, Jiangxi, PR China; 2 Department of Health of Jiangxi Province, Nanchang, Jiangxi, PR China

## Abstract

**Background:**

Lumbar disc herniation is a common and costly problem. Moxibustion is employed to relieve symptoms and might therefore act as a therapeutic alternative. Many studies have already reported encouraging results in heat-sensitive moxibustion for lumbar disc herniation. Hence, we designed a randomized controlled clinical trial to investigate the effectiveness of heat-sensitive moxibustion compared with conventional moxibustion.

**Methods:**

This trial is a multicenter, prospective, randomized controlled clinical trial. The 316 eligible patients are randomly allocated to two different groups. The experimental group is treated with heat-sensitive moxibustion (n = 158); while the control group (n = 158) is treated with conventional moxibustion. The moxibustion locations are different for the groups. The experimental group selects heat-sensitization acupoints from the region which consists of bilateral Da Changshu (BL25) and Yao Shu (Du2). Meanwhile, fixed acupoints are used in control group; patients in both groups receive 18 sessions in 2 weeks.

**Discussion:**

The study design guarantees a high internal validity for the results. It is one large-scale randomized controlled trial to evaluate the efficacy of heat-sensitive moxibustion compared to conventional moxibustion and may provide evidence for this therapy as a treatment for moderate and severe lumbar disc herniation. Moreover, the result may uncover the inherent laws to improve the therapeutic effect with suspended moxibustion.

**Trial Registration:**

The trial is registered at Chinese Clinical Trials Registry: ChiCTR-TRC-09000604. The application date was 27 November 2009. The first patient was randomized on the 16 June 2011.

## Background

Lumbar disc herniation (LDH) is one of the most common causes of nerve root pain, and become a painful, debilitating disorder in working adults [1]. In the majority of patients, experiencing their first episode of sciatica due to LDH, the symptoms recede to a non-disabling level within a period of six weeks [2].

LDH most typical symptoms are low back pain, accompanied by one or both lower extremity pain, numbness, pain, mostly by lumbosacral, buttocks, posterolateral to the lower limbs and feet, back radiation when bending, cough worse with movements. Herniated discs also often occur without symptoms, as revealed by magnetic resonance imaging studies in asymptomatic people. They are only clinically relevant when they impinge on a nerve root, causing radiculopathy [3,4].

In general, LDH is due to disc degeneration or trauma leading to nucleus, annulus fibrosis to the vertebral canal prominent, spinal cord or nerve root compression. As a result, conventional treatments including physical therapy, pain medication, anti-inflammatory drugs and surgery, are provided by physicians. Most patients with low back pain respond well to conservative therapy [5,6]. Recently, a large number of patients with LDH are turning to complementary and alternative treatments. Complementary and alternative treatments such as acupuncture are therefore attractive. Acupuncture is often used for LDH. For example, it is gaining popularity among LDH patients in the US and about 1 million consumers with musculoskeletal disorders utilize acupuncture annually [7].

Moxibustion is a traditional Chinese method that uses the heat generated by burning herbal preparations containing Artemisia vulgaris to stimulate acupoints [8]. Moxibustion has anti-inflammatory or immunomodulatory effects against chronic inflammatory conditions in humans [9,10]. Different moxibustion methods for treatment of LDH and their mechanism may be due to moxibustion can improve local blood circulation, eliminate nerve root inflammation and edema, loosen adhesions and improve protrusion and nerve root relations or the promotion of nerve injury repair [11,12]. Moreover, the heat of moxa treatment improves microcirculation in the lumbar vertebra.

Therefore, these arthritis substances may be reduced and weakened by moxibustion. Especially for acute LDH, moxibustion may have a good effect. Severe low-back pain with leg pain (sciatica) may be caused by a herniated intervertebral disc exerting pressure on the nerve root in this stage. Nerve root inflammation and the surrounding edema aggravate the pain. The role of moxibustion may manage the pathological process.

There are many factors influencing therapeutic effect in moxibustion. But the selection of location for manipulating moxa should deserve the greatest attention [13]. Our previous studies suggested that the dominating factor in selecting the location of acupoints is associated with the area on the body surface which is affected by disease, not only the standardized fixed position [14]. In humans, acupoints contain two states: stimulated and resting. When people get sick, the acupoints on the body surface area are activated and sensitized. The sensitive areas are susceptible to heat stimulation and called "heat-sensitized acupoints". A feature of these areas is that they are specific or closely relevant to acupoints and produce the same clinical effect as "a small stimulation induces a large response". This sensitized acupoint is not only the pathological phenomenon reflecting the diseases but also an effective stimulating location with acupuncture and moxibustion [15].

Our research found that the heat-sensitive phenomenon to acupoint or an area is a new type of reaction in a pathological state [16-19]. A lot of observations and research were used to confirm this phenomenon in the 1990s [16]. We summarized the experiential evidence and found that the state of acupoints might change from the rest state to the heat-sensitized state in patients when suffering from diseases. These special acupoints might bring out further heat sensation and response in the stimulation of moxibustion heat. If we can search out these heat-sensitized acupoints associating with pathological state, good effects may be achieved. Therefore, selecting the heat-sensitized acupoint may obtain therapeutic effect far better than acupuncture and moxibustion at acupoints of the routine resting state [14]. So the heat-sensitive moxibustion is a medical technique usually involving suspended moxibustion on the heat-sensitized acupoint. A great many physicians utilized heat-sensitive moxibustion therapy in different kinds of diseases in China. Moreover, several articles and research have documented the effectiveness and safety of heat-sensitive moxibustion, such as myofascial pain syndrome [18], LDH [20], pressure sores [21] and knee osteoarthritis [22]. Together these findings suggest a superiority effect of heat-sensitized acupoints. However, the results do not provide convincing evidence. This may be because of inappropriate sample size, variability of acupuncture and sham protocols, and missing information which were frequent methodological problems [19]. Therefore, well-designed randomized controlled clinical trials are needed to establish the efficacy of heat-sensitive moxibustion for LDH.

## Method/design

### Objective

The aim of this study is to investigate the effectiveness of heat-sensitive moxibustion compared with conventional moxibustion in patients with acute LDH in China.

### Outcome

The improvement Japanese Orthopedic Association has proposed a series of criteria to define patient response in the context of clinical trials of LDH. M-JOA scale is known as the modified edition of JOA Back Pain Evaluation Questionnaire [23]. According to these criteria, a patient with LDH is assessed including pain, the activities of daily life and work, function impairment, and special exams (Table 1). This scoring system was previously validated [23]. The degree of LDH is divided into three levels: mild: < 10, moderate: 10 to 20, severe: > 20.

**Table 1:** List of M-JOA

Item	Grade/Classification	Score
Subjective symptoms		
Lumbar and leg pain	No	0
	Mild pain or occasional moderate pain	1
	Usual moderate pain or occasional severe pain	2
	Frequent or continuous severe pain	3
Numbness	No	0
	Occasional	1
	Frequent but relief may be on their own	2
	Continuous, not alleviated	3
Objective signs		
Paravertebral tenderness	No	0
	Mild	1
	Moderate	2
	Severe	3
Myodynamia	Grade V	3
	Grade IV	2
	Grade III	1
	Grade 0 to II	0
Straight let raising test	> 70°, straight leg drive up (-)	0
	> 45°, straight leg drive up (+)	1
	> 30°, straight leg drive up (+)	2
	< 30°, straight leg drive up (+)	3
Radiating pain	No	0
	Hip/thigh	1
	Shank	2
	Foot	3
Activities of daily life and work		
Stoop and lift heavy things	Normal stoop, lift weight > 3 kg	0
	Able to stoop, lift weight ≤ 3 kg	1
	Unable to stoop	2
	Serious difficulties in bending and lifting	3
Walking distance/time	Walking ≥ 1 km or 60 min	0
	Walking ≥ 500 m or 30 min	1
	Walking ≥ 100 m or 10 min	2
	Serious difficulties in walking	3
Daily time in bed(h)	< 10	0
	10 to 12	1
	12 to 16	2
	> 16	3
Ability to work	Full-time to do as before	0
	Although able to work, occasionally need a break	1
	Although able to work, usually need a break	2
	Unable to work	3

Therapeutic effect is assessed by comparing baseline and final conditions reported by the patient. This trial also records adverse effects reported by patients during treatment. The outcome measures above will be assessed before treatment, the 14 days after the last moxibustion session and 6 months after the last moxibustion session.

To evaluate the improvement of severity, we will make use of M-JOA score and take the secondary analysis. Improvement rate = [(scores before treatment - after treatment score)/pretreatment score] × 100%. Definition are listed below: Clinically important improvement as ≥ 75%, markedly effective improvement as 50 to 75%, improved as 30 to 50% and ineffective as < 30%. The numbers in the four categories will be calculated respectively at every time point.

### Design

**Figure 1:**
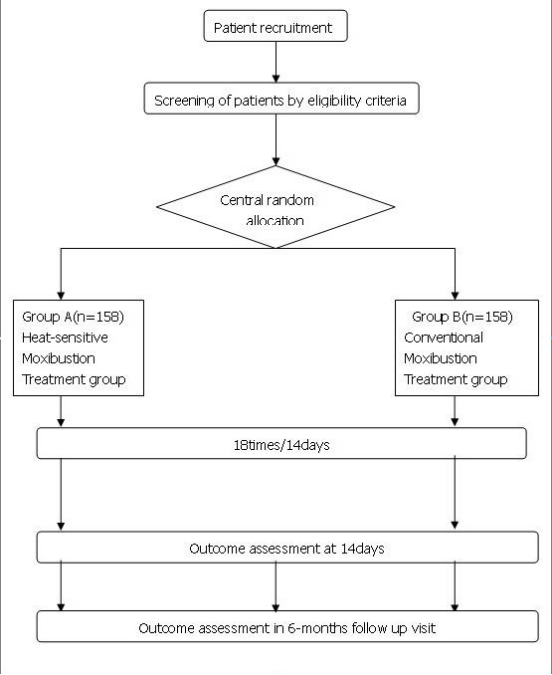
**Test flow chart**.

A multi-center (four centers in China), randomized, subject blinded(group A and B) and assessor blinded, parallel positive controlled trial will be conducted at the affiliated Hospital of Jiangxi University of TCM in Nanchang, The first Affiliated Hospital of Anhui University of TCM in Hefei, Jiangsu TCM Hospital in Nanjing, and Shanxi TCM Hospital in Xian. The study will be sequentially conducted as follows: a recruitment period prior to randomization, a treatment period of 14 days (5 sessions per week), and a follow-up period of six months. Participants will be randomized to the heat-sensitive moxibustion group or the conventional moxibustion group by the central randomization system (Figure 1). This system is provided by China Academy of Chinese Medical Sciences, which adopted the computer telephone integration (CTI) technology to integrate computer, internet and telecom. The random number list will be assigned by interactive voice response (IVR) and interactive web response (IWR) [24]. The success of blinding will be assessed at each participant's last visit. Researchers who did not participate in the treatment and who are blinded to the allocation results will perform the outcome assessment. Patients meeting the inclusion criteria were randomly assigned in a 1:1 ratio to the two groups. We used the parallel controlled design and did not stratify by center or block within center.

### Eligibility

#### Inclusion criteria

Eligible participants will be those previously diagnosed with moderate to severe LDH, according to the M-JOA criteria(> 10 score). Patients will be required to complete the baseline LDH diary. Written informed consent will be obtained from each participant. Participants 18 to 65 years of age will be recruited from outpatient and inpatient in four centers. Standard of diagnosis listed as follows:

① Pain occurred in lower back and radiated to the lower limb; ② Limitations of tender point; ③ Straight leg raising test and it's strengthen test are positive; ④ Skin sensation, muscle strength and tendon reflex have some changes; Changes in spinal posture; ⑤ CT suggestive of disc herniation. The above standard mainly originated from the guiding principle of clinical research on new drugs (GPCRND) [23]. Since the research involves moderate to severe LDH or severe LDH, the inclusion criteria will restrict the following conditions: According to the below LDH diagnosis standard, meanwhile, the triangle region formed with bilateral Da Changshu (BL 25)and Yao Shu (Du 2) of patients (Da Changshu- Yao Shu -contralateral Da Changshu intra-region) appear heat-sensitive points.

Participants will be instructed to stop LDH symptomatic relief medication during the run-in and treatment periods and will be provided the usual care instruction for LDH.

Figure 1 The flow diagram is intended to depict the passage of participants through this RCT.

#### Exclusion criteria

Participants will be excluded if they suffer from serious life-threatening disease, such as disease of the heart and brain, blood, vessels, liver, kidney and hematopoietic system, and psychotic patients. Participants will not be eligible if they are female and whether pregnant or lactating. The following conditions

are also excluded items: appeared a single nerve palsy, or cauda equina nerve palsy, manifested as muscle paralysis or have rectum or bladder problems; complicated with lumbar spinal canal stenosis and space-occupying lesions or for other reasons; complicated with lumbar spine tumors, infections, tuberculosis; moxibustion syncope and unwilling to be treated with moxibustion; do not sign informed consent.

### Treatment protocol

#### Heat-sensitive moxibustion

Moxibustion will be performed by certified acupuncture medical doctors at four centers. Qualified specialists of acupuncture in traditional Chinese medicine with at least five years of clinical experience will perform the acupuncture in this study. All treatment regimens will be standardized between four centers practitioners via video, hands-on training and internet workshops. Participants will be randomly assigned to the heat-sensitive moxibustion group, the conventional moxibustion group. In the former groups, a 22 mm (diameter) × 120 mm (length) moxa-sticks (Jiangxi Traditional Chinese Medicine Hospital, China) will be used. The patient is usually in the comfortable supine position for treatment, with 24°C to 30°C temperature in the room.

For the heat-sensitive moxibustion group, the moxa-sticks are lit by the therapist and held over the region consisting in two Da Changshu (BL 25) and Yao Shu (Du 2) of patients. The warming suspended moxibustion about distance of 3 cm are used to search for the acupoint heat-sensitization phenomenon. The following patient sensation will suggest the special heat-sensitization aupoint: diathermanous sensation due to moxa-heat, defined as the heat sensation conducting from the moxa local skin surface into deep tissue; expand heat sensation due to moxa-heat, defined as the heat sensation spreading to the surrounding little by little around the moxa point; transfer heat sensation due to moxa-heat, defined as the heat sensation transferring along some pathway or direction. When some acupoint exists one below sensation at least, therapists mark the point as a heat-sensitive acupoint. We try our best to seek all the special acupoints in each patient by the repeated assessment.

The therapists begin to treat patients from the most heat-sensitive intensity acupoint. Treatment sessions end when patients feel the acupoint heat-sensitization phenomenon has disappeared. Generally speaking, we select one point each time. One point is treated 45 minutes. Patients receive the treatment for two times daily in the first four days and for one time daily in remaining ten days. The full treatment contains 18 sessions over 14 days.

#### Conventional moxibustion group

Patients assigned to the conventional moxibustion group will receive fixed acupoint moxibustion. Common practices are similar with the first group. The different manipulation is that the therapists carry out warming moxibustion in traditional acupoint, selecting Da Changshu (BL 25), Wei Zhong (BL40) and A-shi Xue. One point is treated 15 minutes a time. The total time is 45 minutes as well. Three points are treated by suspended moxibustion at the same time. In the treatment, the therapists try to give patients the same intensity of the local warm sensation as the experimental group. The sensation of acupoint heat-sensitization phenomenon is not pursued and not avoided in the treatment. Patients receive the treatment for two times daily in the first four days and for one time daily in remaining ten days. The full treatment contains 18 sessions over 14 days.

### Statistical methods

#### Statistical analysis plan

We will conduct analysis on an intention to treat basis, including all randomized participants. Analyses will be conducted using 2-sided significance tests at the 5% significance level. The analysis of center effect will be used logistic regression method. The statistician conducting the analyses will remain blind to treatment group and data will only be unblinded once all data summaries and analyses are completed. All analyses will be conducted in the SAS statistical package program (ver. 9.1.3).

#### Baseline data

Baseline characteristics will be shown as mean, standard deviation (SD) for continuous data including age, previous duration, and so on. The first step of analysis is to check for integrity of randomization. The unbalanced analysis in baseline characteristics will use ANCOVA.

#### Outcome data

These will be summarized descriptively (mean, SD, median, minimum and maximum) at each time point by treatment group. The t-test, Mann-Whitney U and Wilcox on test were used for comparison of variables, as appropriate. When the data do not fit a normal distribution, we will consider more general tests such ANOVA and possible transformations.

All adverse events reported during the study will be included in the case report forms; the rates of adverse events will be calculated. The percentage of subjects with adverse events in each group will be calculated and compared using the chi-squared test or Fisher's exact test. The outcomes will be analyzed using linear mixed and logistic regression models that will include their respective baseline scores as covariates, subjects as a random effect and treatment conditions as fixed factors. Regression diagnostics will be used to check for normality of the measures and homogeneity of variance as appropriate.

##### Dropouts or missing data

Reasons for dropout or missing data will be explored descriptively. Multiple imputation will be used to make sure that dropouts do not impact the conclusions [25].

##### Follow-up data

The primary outcome will be the M-JOA scores. The primary analysis will compare the two groups at 14 days. A secondary analysis will compare the two groups at 6 months to assess if any differences between groups have been maintained over time.

Loss to follow-up is likely to lead to biased estimates of intervention effect. We will try to avoid bias due to attrition by carefully following up the participants in both groups. We will phone participants who do not complete questionnaires after a second reminder. We anticipate a 20% loss to follow-up in this trial, and will implement procedures to minimize loss to follow-up and patient withdrawal, and where possible we will collect information on reasons for patient withdrawal.

#### Data integrity

The integrity of trial data will be monitored by regularly scrutinizing data sheets for omissions and errors. Data will be double-entered and the source of any inconsistencies will be explored and resolved.

#### Sample size

We wished to estimate the sample size that would suffice to detect M-JOA between the heat-sensitive and conventional moxibustion groups. If we apply a two-sided 5% significance level, 90% power the calculated required sample size is approximately 126 participants in each group, according to the equations in [26]. Allowing for a 20% loss to follow up, a total of 158 participants will be required in each group, with 316 participants in total.

### Adverse events

We define adverse events as unfavorable or unintended signs, symptoms or disease occurring after treatment that are not necessarily related to the moxibustion intervention. In every visit, adverse events will be reported by participants and examined by the practitioner.

### Ethics

Written consent will be obtained from each participant. This study was approved by all relevant local ethics review boards. Ethics Committee of Affiliated hospital of Jiangxi Institute of Traditional Chinese Medicine had approved this trial: code issued by ethic committee is 2008(11).

## Discussion

Currently, although different therapeutic methods can be used in patients with LDH, the treatments can be divided into two categories, conservative (or non-surgical) and surgical. Conservative treatments mainly avoid painful positions and relieve symptoms in nine out of 10 people with a herniated disk, according to the American Academy of Orthopedic Surgeons [27]. Many clinical trials held that moxibustion should be effective in the treatment of LDH. But the evidence obtained from these trials was quite limited because of methodology defects. Therefore, we designed this clinical trial to meet the CONSORT statement and guidelines to improve the chances of high internal validity for the results [28]. On the basis of the classical notion of traditional Chinese medicine, moxibustion caused by the burning of moxa leads to the radiant heat and bring drug-like effects to acupoints. Practitioners use moxa to warm acupoints with the intention of stimulating circulation through the acupoints and inducing a smoother flow of blood and qi. So the selection of moxa locations has an important impact on obtaining good effects. It is widely believed that the acupoint locations are fixed along meridians. The conventional moxibustion applies moxa to get the desired results by stimulating these fixed acupoints. Doctors regard hyperemia due to local skin vasodilatation as the indicator of moxibustion effect. However, we support the notion that these fixed acupoints might not be the best stimulating sites for moxibustion.

In humans, acupoints contain two states: stimulated and resting. When people get sick, the acupoints on the body surface area are activated and sensitized. The sensitive areas are susceptible to heat stimulation and are called "heat-sensitized acupoints". Acupoints are more than fixed skin sites but externally sensitive reflecting diseases. Therefore, acupoint is variable and determined by the pathological state. Heat-sensitive moxibustion is a therapeutic method through heat-sensitized acupoints [14].

Acupuncture and moxibustion is a form of therapy derived from Traditional Chinese Medicine (TCM). It is well known that *Huáng Dì Nèi Jīng (黄帝内经) *is the source of theoretical and academic foundation in TCM. According to core theory in this classic,

pathological state of the internal diseases can be manifested through the acupoints. It is very difficult for persons to experience the existence of the acupoints in a normal physical state. However, patients can usually feel some changes in the area of the acupoints when they are sick.

With the state transition and change, sensitized status is often observed. Acupoints on the body surface may be sensitized with various types of sensitization under the circumstances. On the one hand, these sensitized acupoints are supposed to be the pathological phenomena reflecting the diseases; on the other hand, acupuncture and moxibustion treat diseases by stimulating these acupoints [14,15]. Our clinical experience and research in the 1990s pointed out that acupoint

heat-sensitization was a type of acupoint sensitization. The features of acupoints heat-sensitization correspond with the classical thought and theory from the Inner Canon of Huangdi, according to the previous analysis in the above paragraphs [29]. Therefore, it would be valuable to know whether heat-sensitized acupoints is superior to fixed points.

It is hypothesized that selecting the heat-sensitized acupoint may obtain therapeutic effect far better than moxibustion at acupoints of routine resting states. Our purpose is to test and verify the hypothesis. The study outcomes will facilitate the development of the theory of acupuncture and moxibustion. Therefore, the primary aim of this project is more than providing the efficacy of heat-sensitive moxibustion as a treatment modality in patients with LDH. The results of our trial will be helpful to disclose inherent law of moxibustion and present evidence for better therapeutic options to enhance the efficacy of moxibustion in China.

## Competing interests

The authors declare that they have no competing interests.

## Authors' contributions

RC and MC obtained funding for the research project and drafted the protocol. JX wrote the final manuscript. FY contributed to the research design and made critical revisions. ZC and BZ were responsible for the statistical design of the trial and wrote portions of the statistical methods, data handling, and monitoring sections. All authors read and approved the final manuscript.
